# The effect of temporary closure and enhanced terminal disinfection using aerosolized hydrogen peroxide of an open-bay intensive care unit on the acquisition of extensively drug-resistant *Acinetobacter baumannii*

**DOI:** 10.1186/s13756-020-00772-z

**Published:** 2020-07-14

**Authors:** Rima Moghnieh, Hani Tamim, Marwa Jadayel, Dania Abdallah, Rasha Al-Kassem, Hind Kadiri, Hani Hafez, Salam Al-Hassan, Lina Ajjour, Rawad Lakkis, Tamima Jisr, Nadia-Lara Samaha, Nicholas Haddad

**Affiliations:** 1 Division of Infectious Diseases, Department of Internal Medicine, Makassed General Hospital, Beirut, Lebanon; 2 Division of Infectious Diseases, Hôtel Dieu de France, Beirut, Lebanon; 3 Department of Internal Medicine, American University of Beirut, Beirut, Lebanon; 4 School of Pharmacy, Beirut Arab University, Beirut, Lebanon; 5 Pharmacy Department, Makassed General Hospital, Beirut, Lebanon; 6 Nursing Department, Makassed General Hospital, Beirut, Lebanon; 7 Department of Internal Medicine, Makassed General Hospital, Beirut, Lebanon; 8 Faculty of Arts and Sciences, American University of Beirut, Beirut, Lebanon; 9 Department of Laboratory Medicine, Makassed General Hospital, Beirut, Lebanon; 10 College of Human Ecology, Cornell University, Ithaca, NY 14853 USA; 11 Associate Professor of Infectious Disease and Residency Program Director, Internal Medicine, Central Michigan University, Saginaw, MI 48602 USA

**Keywords:** Enhanced terminal disinfection, Hydrogen peroxide, Extensively drug-resistant *Acinetobacter baumannii*, Intensive care unit, Open-bay, Contact pressure, Lebanon

## Abstract

**Background:**

At Makassed Hospital’s open-bay intensive care unit (ICU), enhanced terminal disinfection (ETD) using hydrogen peroxide (H_2_O_2_) was performed without a predefined schedule in extensively-drug-resistant *Acinetobacter baumannii* (XDR-AB) outbreaks. In this study, we aimed to check for the value of the temporary closure of the ICU and the use of ETD with aerosolized H_2_O_2_ and Ag^+^ on minimizing the rate of XDR-AB acquisition in patients admitted to the ICU of our facility, which might consequently help us determine the optimal schedule for such procedure in this unit.

**Methods:**

This is a retrospective medical file review of patients admitted to the ICU between January 2016 and May 2018. We divided this period into numerical weeks (NW) after each closure and ETD episode. Risk factors of acquisition (RFA) were determined by comparing the characteristics of patients who acquired XDR-AB to those who didn’t. The proportion of patients residing in each NW was included in the RFA analysis.

**Results:**

Out of 335 patients, 13% acquired XDR-AB. The overall incidence of XDR-AB acquisition was 14.6 cases/1000 patient days. RFA were XDR-AB contact pressure ≥ 3 days [Odds Ratio (OR) = 9.86, 95% Confidence Interval (CI) (3.65–26.64), *P* < 0.0001)], mechanical ventilation [OR = 4.99, 95%CI (1.76–14.15), *P* = 0.002)], and having a wound [OR = 3.72, 95%CI (0.99–13.96), *P* = 0.05)]. Patients who stayed during NW 7,11 and 14 were at risk of acquisition where the odds significantly increased by 6.5, 9.7 and 14.4 folds respectively (*P* = 0.03,0.01, and 0.01, respectively). We considered NW 7 as the most suitable time for temporary closure of the ICU and ETD with aerosolized H_2_O_2_.

**Conclusion:**

Contact pressure, mechanical ventilation, and presence of a wound were RFA of XDR-AB. Temporary closure of the ICU with ETD using aerosolized H_2_O_2_ decreased the rate of XDR-AB acquisition, yet this effect fades away with time. The ETD was shown to be most efficiently done when repeated every 7 calendar weeks in our open-bay ICU as part of a prevention bundle.

## Background

With the turn of the century, the upsurge of antimicrobial resistance (AMR) has become a worldwide public health threat and its control has become a global priority [1]. Lebanon is at risk for this alarming situation where carbapenem-resistant Gram-negative organisms are widespread in hospitals and have reached the community setting [2–5]. Some of these organisms are even resistant to almost all available antibiotics [6, 7]. Consequently, controlling their spread in both settings is of critical importance.

*Acinetobacter baumannii* is one of the most troublesome pathogens in hospitals causing nosocomial infections and it is associated with high mortality rates, especially among critically ill patients [8, 9]. Of particular concern is its prolonged survival in the hospital environment through its resistance to desiccation thus causing sustained outbreaks [10]. Its inherent and acquired mechanisms of resistance to multiple antibiotics render it a difficult-to-treat pathogen [8, 9]. Carbapenem-resistant or extensively-drug resistant *Acinetobacter baumannii* (XDR-AB) is already endemic in many hospitals in Lebanon [2–6] and pan-drug resistant isolates that are resistant to colistin are progressively appearing in hospitals [7, 11].

Transmission of A. baumannii from the inanimate hospital environment to patients has been well documented [10, 12, 13]. As part of an infection prevention bundle, enhanced terminal environmental disinfection (ETD) is one of the modalities that have been effective in decreasing the spread of these organisms [14]. The most common non-touch techniques of ETD are using ultraviolet light or hydrogen peroxide vapor or aerosol [15].

Makassed General Hospital (MGH), a tertiary care hospital in Beirut, has an open-bay intensive care unit (ICU). As of 2011, XDR-AB acquisition has been reported at an average of 1 case per 80 patient days in this unit (non-published hospital surveillance data). ETD using aerosolized hydrogen peroxide (H_2_O_2_) and Silver ions (Ag^+^) is performed in this ICU and in rooms of the hospital that have been previously occupied by patients colonized or infected patients with pan-drug and extensively-drug resistant bacteria, amongst which is A. baumannii. In the ICU, there is no predefined schedule for ETD where it is performed upon the request of physicians. It is also subject to convenience, based on bed occupancy as ETD necessitates evacuation of the unit for at least 24 h. In this study, we primarily aimed to check for the value of temporary closure of the ICU along with ETD using aerosolized H_2_O_2_ and Ag^+^ on minimizing the rate of XDR-AB acquisition in patients admitted to the ICU of our facility, which might consequently help us determine the optimal schedule for such procedure in this unit as part of an infection prevention bundle. A secondary endpoint was to check for the presence of risk factors of XDR-AB acquisition, related to patient clinical characteristics, the use of invasive devices, and the use of antimicrobial agents. Risk factor identification and mitigation are critical pillars in decreasing the transmission of XDR-AB and these factors will be additionally controlled for in the analysis model.

## Methods

### Setting and study design

This is a retrospective study conducted at MGH, a 186-bed university hospital and tertiary care referral center, with a 5-bed open-bay mixed medical and surgical adult ICU. The study period extended from January 2016 to May 2018. It included data from patient charts admitted to the ICU during the study period. Clinical, microbiological and radiographic information were retrieved from electronic medical records. Other information regarding the number of admissions to the ICU, bed census and number of patient days (PD) were obtained from the nursing head office. The dates of terminal cleaning during the study were retrieved from the hospital administration records. The hospital’s Institutional Review Board committee approved this study and patient consent was waived due to its retrospective nature.

### Infection control considerations

Active microbiological screening for patients’ colonization status and acquisition of carbapenem-resistant organisms was performed during the study period in the ICU. Specimens from the throat, axillae, urine, and perineal area were routinely cultured, in addition to sputum and/or skin lesion (aspirate/biopsy/swab) when applicable. These cultures were secured upon ICU admission and on a weekly basis, as long as the patient was still there and whenever the clinical situation necessitated. The nurse-to patient ratio in the ICU was 2 registered nurses and 3 practical nurses to 5 patients (2:3:5).

Infection control measures were applied according to the Centers for Disease Control and Prevention (CDC) guidelines for the control of spread of extensively-drug resistant organisms in healthcare settings [16]. Daily ICU environmental cleaning and disinfection, with the beds occupied, was performed using an alcohol-based disinfectant and a quaternary ammonium containing disinfectant. As mentioned previously, ETD was performed after evacuating the unit according to bed occupancy convenience, the ICU/Infectious Disease physician request and/or Infection Control team recommendations, without a predefined schedule. It was performed using 6% aerosolized H_2_O_2_ with 60 part-per-million Silver ions (Ag^+^) (BioGienie™ H1000 Version 1, HHYGIENICS™ BIOSECURITY, Hampshire, United Kingdom).

### Microbiological identification

The identification of bacteria from all types of cultures was performed according to standard microbiological procedures. Surveillance cultures targeted carbapenem-resistant Gram-negative bacilli, such as *A. baumannii,* by using MacConkey agar with a 10 μg ertapenem disc and a 10 μg meropenem disc. All microbiological methods were consistent with the Clinical and Laboratory Standards Institute (CLSI) guidelines and antimicrobial susceptibility was determined using the CLSI breakpoints of each corresponding year [17]. XDR-AB was considered resistant to at least one agent in all but two or fewer antimicrobial categories (i.e. bacterial isolates remain susceptible to only one or two categories) [18]. In this study, XDR-AB remained susceptible to polymyxin E (colistin) and tigecycline.

### Grouping of patients

In this study, we divided ICU patients to three groups based on their acquisition of XDR-AB:

- Group 1 or test group included patients who acquired XDR-AB during their stay at least 48 h after admission to the ICU or until 1 week after discharge from the ICU. This acquisition was defined as the isolation of the organism for the very first time from any body site in the patient’s history. All subsequent positive XDR-AB cultures from the same patient were considered duplicate and were excluded from the analysis.
-Group 2 or control group included patients admitted to ICU during the study period and who did not show any evidence of XDR-AB acquisition at anytime.
-Group 3 included patients who:

1. Had positive XDR-AB cultures recovered from any body site within the first 48 h of admission to the ICU,
2. Had evidence of colonization and /or infection with XDR-AB from a previous admission to our facility during 1 year prior to the study period,
3. Were admitted to our ICU or any other ICU in the past 3 months prior to the study period,
4. Were transferred from or admitted to another acute-care or long-term healthcare facility within the past 3 months prior to the study period.

### Potential risk factors

Patients in Groups 1 and 2 only were included in the analysis of risk factors for XDR-AB acquisition. The parameters in question were age, gender, reason for ICU admission, history of intra-abdominal surgery, mechanical ventilation and its duration, use of bilevel positive airway pressure (BiPAP), central venous catheterization and its duration, urinary catheterization and its duration, use of other types of catheters with the duration, presence of wounds or pressure ulcers, the use of vasopressors, the needs of blood transfusion, duration of ICU stay, Acute Physiologic Assessment and Chronic Health Evaluation II (APACHE II) score, Charlson Comorbidity Index (CCI) score, carbapenem and/or piperacillin tazobactam intake with the duration, and XDR-AB contact pressure days. For each patient, contact pressure is a way to measure the extent of contact with colonized or infected patients during a certain bed occupancy time, that is to determine the threat of XDR-AB acquisition exerted by other colonized or infected patients. Contact pressure, expressed in days, is calculated by adding up the number of days that were spent in the vicinity of a colonized or infected patient, i.e. in the same ICU bay or sharing the same room outside ICU in this study [2]. Patients in Group 3 were not included in the risk factor analysis; however, they were included in the calculation of XDR-AB contact pressure days for each patient in Groups 1 and 2. For all time-dependent parameters in Group 1, we only took into account the duration preceding the date of XDR-AB acquisition.

### The effect of temporary closure and ETD using aerosolized H_2_O_2_ and ag^+^ on XDR-AB acquisition

We obtained the dates of ETD from the IC department, and, accordingly, we divided the study period into numerical week, following each event of ETD. The first week after each ETD was considered ‘Week 1’, followed by ‘Week 2’, etc. In order to determine the risk of XDR-AB acquisition in patients residing in the ICU during each numerical week, we recorded the number of patients who stayed in the ICU in each week as per their admission and discharge dates. Patients were counted more than once depending on their duration of stay with respect to the numerical weeks after ETD. The numerical weeks were considered potential risk factors to be included in the statistical analysis between Groups 1 and 2. Patients in Group 3 were excluded from this analysis.

### Incidence of XDR-AB acquisition with time (numerical weeks after ETD)

The number of PD in the ICU was calculated by counting the patient census at the same time each day. We plotted the number of PD (Y-axis) against each numerical week (‘Week 1, 2, 3, etc.’) (X-axis). We also plotted the incidence rate of XDR-AB acquisition in Group 1 (the number of patients who acquired XDR-AB per 1000 PD) (Y-axis) against each numerical week (X-axis), taking into account their first acquisition. Each patient was counted once per each numerical week depending on the date of acquisition only and it was not cumulative. Only the numerical weeks with PD > 50 were included in the plot of XDR-AB incidence and in the analysis of the relation between the numerical week after ETD and the probability of acquiring XDR-AB in ICU.

### Statistical analysis

Statistical analysis was performed by the Statistical Package for the Social Sciences program (SPSS) version 24 (SPSS, Chicago, IL, USA). All p-values were two tailed and *P* < 0.05 was considered statistically significant. Categorical variables were compared to assess any significance using chi-square test and were presented as frequency (%). Bivariate analysis of all studied parameters was performed between groups 1 and 2 and those with *P* < 0.05 were included in the multivariate logistic regression. Odds ratio (OR) and 95% confidence interval (CI) were reported to indicate the impact and significance of each variable in the multivariate model. The first numerical week (PD > 50) where there was a statistically significant difference between the groups 1 and 2 in the bivariate model was recorded. This week and all numerical weeks thereafter were included in the multivariate analysis of independent risk factors for XDR-AB acquisition, while controlling for other factors. In the multivariate analysis, the first numerical week with an OR > 2 and a *p*-value < 0.05 was considered the optimal week to routinely perform ETD.

## Results

### Description of patient characteristics

During the study period, 335 patients were admitted to ICU, with a total of 3083 patient days. The baseline clinical characteristics of the study population, and the studied risk parameters are listed in Table 1. The majority of the patients were above 75 years of age (154/335 patients, 46%) and 51% were males (170/335). The two most common causes of ICU admission were respiratory disorders and sepsis (34.9 and 30.4%, respectively). One third of the patients had an APACHE II score of 20 and above and 72.8% had a CCI score more than or equal to 4. Mechanical ventilation was required in 50.4% of the cases, 31.3% of the patients had central lines and 91.3% had urinary catheters. Vasopressors were administered in 51.6% of the patients and blood transfusion in 29.3%. Carbapenems were prescribed in 58.2% of the cohort and piperacillin-tazobactam in 35.5%. The distribution of patients per group was as such: 13.1% for Group 1 (44/335 patients), 84.2% for Group 2 (282/335 patients) and 2.7% for Group 3 (9/335 patients) (Table 1).

**Table 1:** Bivariate analysis of risk factors for extensively drug-resistant *Acinetobacter baumannii* acquisition in the open-bay intensive care unit during the study period

Characteristics	Total (***N*** = 335 patients) (%)	Group 1 (***N*** = 44 patients) (%)	Group 2 (***N*** = 282 patients) (%)	***P*** (Group 1 vs. Group 2)	Group 3 (***N*** = 9 patients) (%)
**Age (years)**					
*≤ 45*	49 (14.6)	4 (9.1)	44 (15.6)	0.23	1 (11.1)
*> 45 and < 75*	132 (39.4)	15 (34.1)	115 (40.8)		2 (22.2)
*≥ 75*	154 (46.0)	25 (56.8)	123 (43.6)		6 (66.7)
**Gender**				0.29	
*Female*	165 (49.3)	25 (56.8)	136 (48.2)		4 (44.4)
*Male*	170 (50.7)	19 (43.2)	146 (51.8)		5 (55.6)
**Cause of ICU admission**					
*Respiratory problem*	117 (34.9)	15 (34.1)	97 (34.4)	0.97	5 (55.6)
*Septic shock*	102 (30.4)	16 (36.4)	83 (29.4)	0.35	3 (33.3)
*Post-surgical Observation*	38 (11.3)	5 (11.4)	33 (11.7)	0.95	0 (0.0)
*Other causes*	78 (23.3)	8 (18.2)	69 (24.5)	0.36	1 (11.1)
**Intra-abdominal surgery**	23 (6.9)	4 (9.1)	19 (6.8)	0.53	0 (0.0)
**Pressure ulcer**	51 (15.2)	9 (20.5)	38 (13.5)	0.22	4 (44.4)
**Wound**	17 (5.1)	6 (13.6)	10 (3.5)	0.01	1 (11.1)
**Urinary catheter**	306 (91.3)	44 (100.0)	253 (89.7)	0.02	9 (100.0)
*< 5 d*	176 (52.5)	10 (22.7)	163 (57.8)	< 0.0001	3 (33.3)
*≥ 5 d*	159 (47.5)	34 (77.3)	119 (42.2)		6 (66.7)
**Mechanical ventilation**	169 (50.4)	36 (81.8)	126 (44.7)	< 0.0001	7 (77.8)
*< 2 d*	204 (60.9)	9 (20.5)	190 (67.4)	0.002	5 (55.6)
*≥ 2 d*	131 (39.1)	35 (79.5)	92 (32.6)		4 (44.4)
**BiPAP use at hospital**	83 (24.8)	11 (25.0)	70 (24.8)	0.98	2 (22.2)
**BiPAP use at home**	25 (7.5)	2 (4.5)	23 (8.2)	0.55	0 (0.0)
**Central line**	105 (31.3)	19 (43.2)	82 (29.1)	0.06	4 (44.4)
**Kemal catheter**	33 (9.9)	6 (13.6)	27 (9.6)	0.42	0 (0.0)
**Hemodialysis in ICU**	44 (13.1)	5 (11.4)	39 (13.8)	0.66	0 (0.0)
**Use of vasopressors**	173 (51.6)	27 (61.4)	141 (50.0)	0.16	5 (55.6)
**APACHE II score**					
*< 20*	220 (65.7)	27 (61.4)	189 (67.0)	0.46	4 (44.4)
*≥ 20*	115 (34.3)	17 (38.6)	93 (33.3)		5 (55.6)
*Between 20 and 29*	86 (25.7)	15 (34.1)	68 (73.1)	0.35	3 (33.3)
*≥ 30*	29 (8.7)	2 (4.5)	25 (26.9)		2 (22.2)
**CCI score**					
*0*	38 (11.3)	3 (6.8)	34 (12.1)	0.047	1 (11.1)
*1–2*	31 (9.3)	4 (9.1)	26 (9.2)		1 (11.1)
*3–4*	75 (22.4)	12 (27.3)	61 (21.6)		2 (22.2)
*5–6*	97 (29.0)	19 (43.2)	76 (27.0)		2 (22.2)
*7–8*	62 (18.5)	6 (13.6)	55 (19.5)		1 (11.1)
*> 8*	32 (9.6)	0 (0.0)	30 (10.6)		2 (22.2)
*< 4*	91 (27.2)	12 (27.3)	76 (27.0)	0.96	3 (33.3)
*≥ 4*	244 (72.8)	32 (72.7)	206 (73.0)		6 (66.7)
**XDR-AB contact pressure**	147 (43.9)	33 (75.0)	113 (40.1)	< 0.0001	1 (11.1)
*< 3 d*	252 (75.2)	14 (31.8)	230 (81.6)	< 0.0001	8 (88.9)
*≥ 3 d*	83 (24.8)	30 (68.2)	52 (18.4)		1 (11.1)
**Blood Transfusion**	98 (29.3)	11 (25.0)	82 (29.1)	0.58	5 (55.6)
**CAR use**	195 (58.2)	31 (70.5)	157 (55.7)	0.06	7 (77.8)
*< 4 d*	208 (62.1)	19 (43.2)	185 (65.6)	0.004	4 (44.4)
*≥ 4 d*	127 (37.9)	25 (56.8)	97 (34.4)		5 (55.6)
*< 7 d*	246 (73.4)	25 (56.8)	216 (76.6)	0.005	5 (55.6)
*≥ 7 d*	89 (26.6)	19 (43.2)	66 (23.4)		4 (44.4)
**TZP use**	119 (35.5)	23 (52.3)	93 (33.0)	0.01	3 (33.3)
*< 4 d*	275 (82.1)	34 (77.3)	234 (83.0)	0.36	7 (77.8)
*≥ 4 d*	60 (17.9)	10 (22.7)	48 (17.0)		2 (22.2)
**CAR or TZP use**	259 (77.3)	43 (97.7)	207 (73.4)	< 0.0001	9 (100.0)
*< 4 d*	164 (49.0)	12 (27.3)	149 (52.8)	0.002	3 (33.3)
*≥ 4 d*	171 (51.0)	32 (72.7)	133 (47.2)		6 (66.7)
**LOS in the ICU**					
*< 4 days*	175 (52.2)	7 (15.9)	164 (58.2)	< 0.0001	4 (44.4)
*≥ days*	160 (47.8)	37 (84.1)	118 (41.8)		5 (55.6)
**Number of patients who stayed in the ICU during each numerical week after ETD**^**a**^					
*Week 1*	54 (16.1)	6 (13.6)	44 (15.6)	0.74	4 (44.4)
*Week 2*	35 (10.4)	5 (11.4)	27 (9.6)	0.78	3 (33.3)
*Week 3*	37 (11.0)	5 (11.4)	32 (11.3)	1.00	0 (0.0)
*Week 4*	35 (10.4)	5 (11.4)	29 (10.3)	0.79	1 (11.1)
*Week 5*	38 (11.3)	6 (13.6)	31 (11.0)	0.61	1 (11.1)
*Week 6*	31 (9.3)	8 (18.2)	21 (7.4)	0.04	2 (22.2)
*Week 7*	30 (9.0)	7 (15.9)	21 (7.4)	0.08	2 (22.2)
*Week 8*	38 (11.3)	6 (13.6)	31 (11.0)	0.61	1 (11.1)
*Week 9*	36 (10.7)	8 (18.2)	26 (9.2)	0.11	2 (22.1)
*Week 10*	19 (5.7)	5 (11.4)	14 (5.0)	0.15	0 (0.0)
*Week 11*	25 (7.5)	7 (15.9)	17 (6.0)	0.03	1 (11.1)
*Week 12*	26 (7.8)	8 (18.2)	17 (6.0)	0.01	1 (11.1)
*Week 13*	22 (6.6)	7 (15.9)	14 (5.0)	0.01	1 (11.1)
*Week 14*	20 (6.0)	10 (22.7)	10 (3.5)	< 0.0001	0 (0.0)
*Week 15*	17 (5.1)	8 (18.2)	9 (3.2)	0.001	0 (0.0)
*Week 16*	18 (5.4)	6 (13.6)	12 (4.3)	0.02	0 (0.0)

*KEY = APACHE* Acute Physiology And Chronic Health Evaluation, *BiPAP* Bilevel Positive Airway Pressure, *CAR* Carbapenems, *CCI* Charlson Comorbidity Index, *d* days, *ETD* Enhanced Terminal Disinfection, *ICU* Intensive Care Unit, *LOS* Length of Stay, *TZP* Piperacillin/Tazobactam, *XDR-AB* Extensively-drug resistant *Acinetobacter bumannii*

**N.B**

In this study, we divided ICU patients to three groups based on their acquisition of *XDR A. baumannii* (XDR-AB):

-Group 1 or test group included patients who acquired XDR-AB during their stay at least 48 h after admission to the ICU or until 1 week after discharge from the ICU.

-Group 2 or control group included patients admitted to ICU during the study period and who did not show any evidence of XDR-AB acquisition at anytime.

-Group 3 included patients who:

1. Had positive XDR-AB cultures recovered from any body site within the first 48 h of admission to the ICU,

2. Had evidence of colonization and /or infection with XDR-AB from a previous admission to our facility during 1 year prior to the study period,

3. Were admitted to our ICU or any other ICU in the past 3 months prior to the study period,

4. Were transferred from or admitted to another acute-care or long-term healthcare facility within the past 3 months prior to the study period.

^a^Patients who resided in the ICU during numerical weeks 1 to 16 were only included since the number of patient days per week was > 50 days. Patients were counted more than once depending on their duration of stay with respect to the numerical weeks after ETD

### Effect of temporary closure and ETD on XDR-AB acquisition measured by the variation in its incidence rate during the numerical weeks

**Fig. 1:**
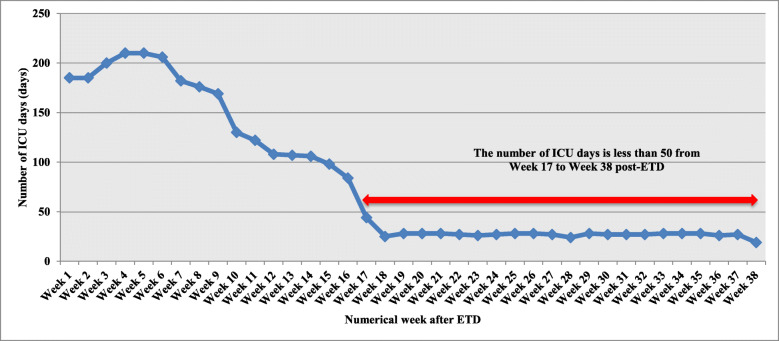
The number of patient days in the open-bay intensive care unit (ICU) of our facility per each numerical week after enhanced terminal disinfection (ETD) during the study period

**Fig. 2:**
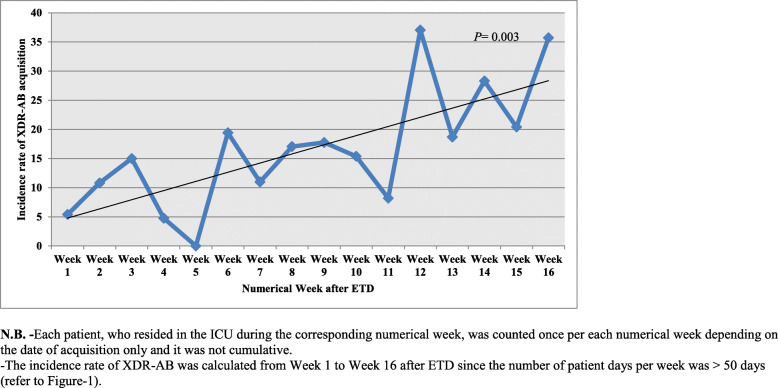
Incidence rate of extensively-drug resistant *Acinetobacter baumannii* (XDR-AB) acquisition (number of cases who had a positive culture per 1000 patient days) in the open-bay intensive care unit (ICU) of our facility per each numerical week after enhanced terminal disinfection (ETD). **N.B. -**Each patient, who resided in the ICU during the corresponding numerical week, was counted once per each numerical week depending on the date of acquisition only and it was not cumulative. -The incidence rate of XDR-AB was calculated from Week 1 to Week 16 after ETD since the number of patient days per week was > 50 days (refer to Figure-1).

Temporary closure of ICU and ETD using aerosolized H_2_O_2_ and Ag^+^ was performed 8 times during the study period and the number of numerical weeks reached 38 (Fig. 1). During the entire study period, the overall incidence of XDR-AB acquisition reached 14.6 cases per 1000 PD. Regarding the variation in the incidence rate of XDR-AB acquisition per numerical week (Fig. 2), a linear increasing trend was observed from numerical Week 1 to Week 16 after ETD, where it was 5.4 cases/1000 PD in Week 1, 11 cases/1000 PD in Week 7, and it significantly increased to 35.7 cases/1000 PD in Week 16 (*P* = 0.003). Weeks 17 to 38 after ETD were not included due to the low number of PD (< 50 days).

### Risk factors for the acquisition of XDR-AB

Through bivariate analysis, there was a statistically significant difference in several parameters between Groups 1 and 2, respectively, thus favoring XDR-AB acquisition in the following: Presence of a wound (13.6% vs. 3.5%, *P* = 0.01), mechanical ventilation (81.8% vs. 44.7%, *P* < 0.0001), placement of a urinary catheter for more than or equal to 5 days (79.5% vs. 32.6%, *P* = 0.002), receipt of carbapenems for more than or equal to 4 days (56.8% vs. 34.4%, *P* = 0.004), receipt of piperacillin-tazobactam (52.3% vs. 33%, *P* = 0.01), ICU stay of 4 or more days (84.1% vs. 41.8%, *P* < 0.0001), and XDR-AB contact pressure of 3 or more days (68.2% vs. 18.4%, *P* = 0.0001) (Table 1).

After controlling for confounding factors, the multivariate model showed that mechanical ventilation increased the risk of XDR-AB acquisition by almost 5 times [OR = 4.99, 95% CI (1.76–14.15), *P* = 0.002)] (Table 2). Patients with XDR-AB contact pressure for 3 or more days were at the highest risk of acquisition [OR = 9.86, 95% CI (3.65–26.64), *P* < 0.0001)]. On the other hand, the presence of a wound increased the risk of acquisition by 4 times and nearly reached statistical significance [OR = 3.72, 95% CI (0.99–13.96), *P* = 0.05)]. Likewise, the receipt of piperacillin-tazobactam and carbapenems for more than 4 days increased the odds of XDR-AB acquisition by almost 3 times yet did not reach statistical significance [OR = 2.86, 95% CI (0.97–8.46), *P* = 0.06); OR = 2.5, 95% CI (0.43–14.36), *P* = 0.31); respectively]. As for the placement of a urinary catheter for 5 or more days and ICU LOS of 4 or more days, both parameters increased the odds by 1.3 folds yet without statistical significance [OR = 1.42, 95% CI (00.42–4.79), *P* = 0.58); OR = 1.29, 95% CI (0.39–4.30), *P* = 0.68); respectively] (Table 2).

**Table 2-:** Multivariate logistic regression of potential predictors for extensively drug-resistant *Acinetobacter baumannii* acquisition during the study period

Variables	Odds Ratio (95% Confidence Interval)	***P***
Wound	3.72 (0.99–13.96)	0.05
Urinary catheter (≥ 5 d)	1.42 (0.42–4.79)	0.58
Mechanical ventilation	4.99 (1.76–14.15)	0.002
CCI score		
*0*	0.71 (0.13–3.98)	0.70
*5–6*	2.55 (0.96–6.81)	0.06
*7–8*	0.48 (0.11–2.14)	0.34
XDR-AB contact pressure (≥ 3 d)	9.86 (3.65–26.64)	< 0.0001
CAR use (≥ 4 d)	2.50 (0.43–14.36)	0.31
TZP use	2.86 (0.97–8.46)	0.06
CAR or TZP use (≥ 4 d)	0.58 (0.13–2.68)	0.49
LOS in the ICU (≥ 4 d)	1.29 (0.39–4.30)	0.68
Number of patients staying in the ICU during each numerical week after ETD		
*Week 6*	3.37 (0.89–12.71)	0.07
*Week 7*	6.47 (1.21–34.50)	0.03
*Week 8*	0.30 (0.04–2.39)	0.26
*Week 9*	3.23 (0.59–17.75)	0.18
*Week 10*	0.12 (0.01–1.03)	0.053
*Week 11*	9.73 (1.66–57.12)	0.01
*Week 12*	2.65 (0.49–14.52)	0.26
*Week 13*	0.14 (0.02–1.24)	0.08
*Week 14*	14.37 (1.75–118.17)	0.01
*Week 15*	1.34 (0.11–15.85)	0.82
*Week 16*	3.33 (0.26–43.02)	0.36

Variables included in the model: Urinary catheter (reference: < 5 d); Mechanical ventilation (reference: no); CCI score; XDR-AB contact pressure (reference: < 3 d); CAR (reference: < 4 d); TZP use (reference: no); CAR or TZP use (reference: < 4 d); LOS in the ICU (Reference: < 4 d); Number of patients staying in the ICU during each numerical week after ETD: Week 6 to Week 16 (reference: no)

*KEY = CAR* Carbapenems, *CCI* Charlson Comorbidity Index, *d* days, *ETD* Enhanced Terminal Disinfection, *ICU* Intensive Care Unit, LOS: Length of Stay, *TZP* Piperacillin/Tazobactam, *XDR-AB* Extensively-drug resistant *Acinetobacter bumannii*

### Numerical weeks after ETD as a potential risk factor of XDR-AB acquisition

By bivariate analysis, Week 6 after ETD was the first to show a statistically significant difference between groups 1 and 2. Accordingly, Weeks 6 to 16 were included as potential risk factors for XDR-AB acquisition in the multivariate logistic regression. Weeks 17 to 38 after ETD were not included due to the low number of PD (< 50 days). The multivariate model showed that patients who stayed during numerical weeks 7,11 and 14 after ETD were at an increased risk of acquisition where the odds significantly increased by 6.5, 9.7 and 14.4 folds respectively (*P* = 0.03, 0.01, and 0.01, respectively) (Table 2). We considered numerical Week 7 after ETD as the most suitable time for ETD since it was the first numerical week with an OR > 2 (6.5) and a statistically significant p-value (0.03).

## Discussion

Carbapenem-resistant A. baumanni or XDR-AB has been recently recognized by the “World Health Organization (WHO) AMR study group” as a high priority Gram-negative organism for the research and development of new antibiotics, given the limited availability of treatment options, its swiftness in developing AMR, in addition to the elevated rates of mortality due to associated infections [19]. Through current evidence, its resistance to disinfection and its endemicity around the globe has exponentially augmented the problem rendering it the nightmare of Infection Control practitioners when managing sustained outbreaks [20]. Another group of international experts developed the “TOp TEn resistant Microorganisms (TOTEM) in critical care” study group, which aimed to assess the global top priority organisms affecting ICU patients specifically [21]. Carbapenem-resistant A. baumannii was again classified as the top critical organism thus coinciding with the WHO priority list [21].

Many critical care and burn units of acute care facilities in the Middle East and North Africa region have become endemic with XDR-AB [22]. In the current study, out of 335 patients admitted to our ICU, 13% acquired XDR-AB during their stay and the overall incidence reached 14.6 cases/1000 ICU days. Other studies from Lebanon and the region reported different burdens of XDR-AB in critical care units. A prospective study conducted over 7 years form 2007 to 2014 at a large university hospital in Beirut showed that the a rate of XDR-AB colonization pressure was 315.4 cases/1000 ICU patient-days [3]. Another matched case–control study from a specialist hospital in the Kingdom of Saudi Arabia between January and August 2012, assessing potential risk factors for XDR-AB acquisition, showed that the proportion of ICU patients who harbored XDR-AB during their stay reached 33% (66/198 patients) [23]. These figures indicate in our geographical region that XDR-AB is rampant in critical care units.

Infections caused by A. baumanii in general and especially among critically ill patients are associated with high morbidity and mortality [24]. To be able to reduce this high mortality rate, a multidisciplinary approach is essential, including early detection and surveillance for acquisition, strategies for patient risk factor identification, antimicrobial stewardship interventions, and tight adherence to standardized infection control practices [2, 25–28].

Risk factor identification and mitigation are critical pillars in decreasing the transmission of XDR-AB [2]. Our study showed that contact pressure of 3 or more days with other patients who harbored XDR-AB was an independent risk factor for acquisition of the same organism, and it significantly increased the odds of horizontal acquisition by almost 10 folds. This can be explained by the fact that the open-bay ICU is one big room and all bed occupants are like “roommates”. It has been also well documented that patients admitted to ICU beds of prior occupants who harbored bacterial pathogens are at an elevated risk of acquiring the same organism, i.e. vertical transmission of the organism [29]. In a multicenter matched case-control study that evaluated the association between having a prior bed occupant or roommate with a positive culture and subsequent infection with the same organism, the risk of infection increased by 6 folds in case of the prior bed occupant (vertical transmission) and 5 times in case of the roommate (horizontal transmission) [30]. With time, transmission of XDR-AB occurring in both directions, vertical and horizontal, will absolutely increase and one will lead to the other in an open-bay ICU. In this setting, ICU evacuation and ETD have been practiced to potentially stem the vertical transmission and break the vicious cycle.

Our results further showed that patients who were administered carbapenems for more than 4 days or piperacillin-tazobactam were more likely to acquire XDR-AB during their hospital stay. Antimicrobial stewardship programs play an important role in sparing the use of carbapenems and other broad-spectrum antibiotics [27]. Their extensive use has been positively correlated with an increasing incidence of XDR-AB [31], while restricting their consumption has led to an important reduction in its incidence [25, 26, 28]. The proper selection of broad-spectrum antibiotics for the empiric treatment of infections is based on the institutional epidemiology of AMR, along with an appropriate duration and de-escalation of therapy once antibiograms are available.

In general, risk factors for XDR-AB acquisition may vary from one ICU to the other due to differences in applying standard precautions, compliance to hand hygiene, distance between beds, type of ICU whether open-bay or single-room [2, 32]. On the other hand, risk factors including the use of invasive devices, treatment with broad-spectrum antibiotics, length of stay in ICU and contact pressure are commonly reported in several studies [2, 3, 23, 33].

Regarding infection prevention, the presence of robust programs is crucial in order to help curb the spread of XDR-AB throughout healthcare systems. Patient cohorting, improved hand hygiene, regular environmental cleaning and disinfection, in addition to novel non-touch techniques used in ETD have succeeded in reducing nosocomial infection rates and controlling outbreaks of XDR-AB [25, 26]. Contactless automated decontamination technologies include aerosol or vaporized H_2_O_2_ and mobile continuous germicidal ultraviolet light (UV-C). Yet, the success of ETD, which is only one part of the puzzle, depends on human factors such as training and compliance of nursing and environmental services staff, as well as accessibility of all inanimate surfaces in the unit. The quality of surface disinfection is highlighted by a study that proved surface contamination with epidemiologically important pathogens owed to a failure to practice thorough cleaning and disinfection rather than a faulty product or procedure [34].

Based on our findings, XDR-AB acquisition followed an ascending trend as a function of numerical weeks after ETD (*P* = 0.03). This implied that more patients acquired XDR-AB with time after ETD due to the waning effect of the procedure. The complete evacuation of the ICU and admitting patients who are not infected or colonized with XDR-AB decreased the contact pressure during the early numerical weeks after ETD, which is a very strong factor of XDR-AB acquisition as mentioned previously. The effect of the contactless modality of disinfection using aerosolized H_2_O_2_ could be explained as such. First, the physical and biochemical properties of the aerosolized H_2_O_2_ particles enable it to reach and potentially decontaminate surfaces that are usually inaccessible by manual techniques [35–37]. Second, H_2_O_2_ could synergistically improve the effect of other common agents used in manual disinfection, such as quaternary-ammonium containing products (used in our case), when applied to cleaned surfaces with potential residual XDR-AB contamination [35–37].

A study from the United Kingdom investigated the efficacy of terminal disinfection using different operating concentrations of vaporized hydrogen peroxide on methicillin-resistant Staphylococcus aureus (MRSA), Klebsiella pneumoniae and Clostridium difficile persistence on single isolation room surfaces after patient discharge [38]. Investigators artificially contaminated high-frequency-touch surfaces with these organisms, where the sites were sampled with contact plates before and after hydrogen peroxide fumigation [38]. After manual disinfection only, more than 90% of the sites were still contaminated with these organisms, with high bacterial count present on floors, bed control panels, and nurse call buttons [38]. Enhanced disinfection with hydrogen peroxide achieved an approximately 5 log10 reduction in C. difficile spores on contact plates and an approximately 6 log10 reduction in MRSA/K. pneumoniae colony forming units on contact plates in all tested areas [38].

The direct effect of ETD in general was recently elucidated in the Benefits of Enhanced Terminal Room (BETR) Disinfection study, a prospective cluster-randomized crossover trial [14]. This study assessed 3 different enhanced methods of room disinfection (bleach, quaternary ammonium-containing product with disinfecting UV-C, and bleach with UV-C compared to a standard disinfection method with quaternary ammonium-containing product only (control) [14]. Results showed that enhanced methods of disinfection overcame limitations of standard disinfection strategies and thus could be potential strategies to reduce the risk of acquisition of multidrug-resistant organisms and *Clostridium difficile* [14]. This trial has shown the efficacy of using UV-C as a non-touch strategy for enhanced disinfection, but not H_2_O_2_. Limited data are available on the activity of aerosolized H_2_O_2_ based on laboratory findings or evaluation of experimentally contaminated surfaces in hospitals [15]. An aerosolized H_2_O_2_ system was capable of eradicating methicillin-resistant *Staphylococcus aureus* and *A. baumannii* on open hospital room surfaces; however, it was not effective in closed or semiclosed areas like inside a drawer [36]. On the other hand, Blazejewski and colleagues reported results of a prospective crossover study in five medical and surgical ICUs located in a single tertiary care hospital, which examined the impact of ETD using H_2_O_2_ on environmental contamination with multidrug-resistant organisms including imipenem-resistant *A. baumannii* [39]. In this study, target rooms (*n* = 182) underwent routine terminal cleaning with a quaternary ammonium compound and bleach. Then, they were disinfected by either H_2_O_2_ vapor or aerosolized H_2_O_2_ combined with peracetic acid during 6 weeks with a switch to the other method for another 6 weeks [39]. Environmental sampling of 8 high-touch surfaces was performed in each room at 3 time points: (1) after patient discharge, (2) after routine terminal cleaning, and (3) after ETD. First after patient discharge, 15/182 (8%) rooms were contaminated with at least one multi-drug resistant organism [39]. Then, routine terminal cleaning reduced environmental contamination with multi-drug resistant organisms from 8 to 6% (11/182 rooms), albeit insignificantly (*P* = 0.371) [14]. Yet, investigators observed a significant reduction in environmental contamination with multi-drug resistant organisms from 11/182 rooms (6%) (after routine terminal cleaning) to 1/182 rooms (0.6%) after ETD using H_2_O_2_ (*P* = 0.004). Interestingly, both studied techniques (aerosolized and vapor H_2_O_2_) showed similar disinfection efficacy [39]. The evidence provided in the aforementioned studies could be used to interpret our results, through highlighting the disinfection efficacy of ETD with H_2_O_2_, in addition to the effect of decreasing contact pressure after ICU evacuation as mentioned earlier. More robust studies on room decontamination should be performed with specific H_2_O_2_ formulations and related ETD techniques, and these should be compared to other standardized and reliable methods.

As we previously mentioned, ETD necessitated the evacuation and closure of our open-bay ICU for at least 24 h. It was not feasible on a regular basis after the discharge of each infected or colonized patient with XDR-AB from the unit. Such intervention is costly regarding the loss of ICU bed occupancy during the time of unit closure. On the other hand, XDR-AB acquisition exerts an economic burden on the hospital, not to mention the associated morbidity and mortality [40]. Finding the optimal timing for ETD would help prevent sustained transmission on one the hand, and unnecessary evacuation and loss of bed occupancy on the other hand. As per the multivariate regression analysis in our cohort, the first numerical week that considerably increased the odds of XDR-AB acquisition with respect to control was ‘Week 7’ after ETD. The chance that a patient staying in the ICU during ‘Week 7’ to have acquired XDR-AB was 6.5 times significantly higher than not to acquire it. So, we recommend performing ETD with aerosolized H_2_O_2_ every 7 calendar weeks in our open-bay ICU as part of an infection control bundle, in order to limit the increasing rate of XDR-AB acquisition with time.

The increasing risk of XDR-AB acquisition with time after ETD in an open-bay ICU makes this design a questionable situation. Separate rooms or cubicles where ETD can be performed after the stay of each colonized or infected patient might be the preferable architecture of an ICU in the era of virulent XDR organism transmission, along with the paucity of effective antimicrobials [12]. Halaby et al. successfully described the implementation of a single room policy in their ICU compared to a previous open-bay design. This shift resulted in a clear and sustained decrease in the prevalence of the multi-drug resistant Gram-negative bacteria, keeping in mind that no significant changes in variables including bed occupancy and numbers of patient admissions was documented during the study period [32]. In 2018, the revised US guidelines of hospital construction provided a new recommendation that critical care units be designed on a single-room basis except for neonatal ICUs [41]. An exception is provided for renovation of patient rooms or cubicles for single-patient use provided they have a minimum clear floor area of 150 square feet [41].

## Limitations and strengths

One major limitation in this study is that our finding regarding the optimal timing of ETD is specific to our open-bay ICU and may not be applicable to other hospital ICUs. Factors affecting this result are the degree of compliance with hand hygiene requirements, the extent of application of patient isolation and standard precautions policies, nurse-to-patient ratios, bed-to-bed distance, and the endemicity of XDR organisms other than A. baumannii. Nevertheless, an attempt to define the most appropriate timing for ETD in a limited-resource setting is a useful roadmap that would assist other researchers build upon and other practitioners plan ETD schedules in their facilities. On the other hand, our findings highlight the need to shift from an open-bay ICU to individual rooms in this era of XDR organisms. Our retrospective study design is another drawback, where a prospective randomized approach with a crossover design would come up with more reliable results, while controlling for potential confounders. Another limitation is the lack of proof for the activity of the H_2_O_2_ decontamination on the quantitative load of A. baumannii in the inanimate environment like it has been demonstrated for other organisms including K. pneumoniae, MRSA and C. difficile. This opens the door to further research regarding the optimal ways to enhance cleaning and disinfecting surfaces contaminated with A. baumannii.

## Conclusion

Contact pressure, indwelling urinary catheters, mechanical ventilation, presence of a wound along with the use of broad spectrum antimicrobials are risk factors for the acquisition of XDR-AB in our facility. Temporary ICU closure along with ETD using aerosolized H_2_O_2_ and Silver cations decreased the rate of XDR-AB acquisition, yet this effect fades away with time. The ETD was shown to be most efficiently done when repeated every 7 calendar weeks in our open-bay ICU as part of an infection control bundle, in order to limit the increasing rate of XDR-AB acquisition with time while most optimally utilizing costly hospital resources. However, traditional cleaning methods should not be relaxed or abandoned even if ETD is regularly performed, emphasizing on the importance on properly using surface disinfectants to remove biofilms. Isolated patient areas with the possibility of independent enhanced cubicle disinfection after discharge of each colonized or infected patient would preferably replace open-bay ICUs in this era of spreading antibiotic-resistant organisms.

## Data Availability

The data that support the findings of this study are available from Makassed General Hospital but restrictions apply to the availability of these data, which were used under license for the current study, and so are not publicly available. Data are however available from the authors upon reasonable request and with permission of Makassed General Hospital.

## Abbreviations

AMR: Antimicrobial Resistance
APACHE II: Acute Physiologic Assessment and Chronic Health Evaluation II
BiPAP: Bilevel Positive Airway Pressure
CCI: Charlson Comorbidity Index
CDC: Centers for Disease Control and Prevention
CI: Confidence Interval
CLSI: Clinical and Laboratory Standards Institute
ETD: Enhanced Terminal Disinfection
H_2_O_2:_: Hydrogen Peroxide
ICU: Intensive Care Unit
MGH: Makassed General Hospital
NW: Numerical Weeks
OR: Odds Ratio
PD: Patient Days
RFA: Risk Factors of Acquisition
SPSS: Statistical Package for the Social Sciences
TOTEM: TOp TEn resistant Microorganisms
WHO: World Health Organization
XDR-AB: Extensively Drug-Resistant *Acinetobacter baumannii*
